# Suicide by pesticide poisoning in India: a review of pesticide regulations and their impact on suicide trends

**DOI:** 10.1186/s12889-020-8339-z

**Published:** 2020-02-19

**Authors:** Toby Bonvoisin, Leah Utyasheva, Duleeka Knipe, David Gunnell, Michael Eddleston

**Affiliations:** 1 Hull University Teaching Hospitals NHS Trust, Hull, UK; 2 Centre for Pesticide Suicide Prevention, University of Edinburgh, QMRI E3.22a, 47 Little France Crescent, Edinburgh, EH16 4TJ UK; 3 Population Health Sciences Institute, Bristol Medical School, Bristol, UK; 4 Pharmacology, Toxicology & Therapeutics, University/BHF Centre for Cardiovascular Science University of Edinburgh, Edinburgh, UK

**Keywords:** India, Suicide, Poisoning, Pesticides, Endosulfan, Prevention, Means restriction

## Abstract

**Background:**

Pesticide self-poisoning is a common means of suicide in India. Banning highly hazardous pesticides from agricultural use has been successful in reducing total suicide numbers in several South Asian countries without affecting agricultural output. Here, we describe national and state-level regulation of highly hazardous pesticides and explore how they might relate to suicide rates across India.

**Methods:**

Information on pesticide regulation was collated from agriculture departments of the central government and all 29 state governments (excluding union territories). National and state-level data on suicides from 1995 to 2015 were obtained from the National Crime Records Bureau (NCRB). We used joinpoint analysis and negative binomial regression to investigate the trends in suicide rates nationally and in Kerala, in view of the robust measures Kerala has taken to restrict a number of HHPs, to identify any effect on suicides.

**Results:**

As of October 2019, 318 pesticides were registered for use in India, of which 18 were extremely (Class Ia) or highly (Class Ib) hazardous according to World Health Organization toxicity criteria. Despite many highly hazardous pesticides still being available, several bans have been implemented during the period studied. In our quantitative analyses we focused on the permanent bans in Kerala in 2005 (of endosulfan) and 2011 (of 14 other pesticides); and nationally in 2011 (of endosulfan). NCRB data indicate that pesticides were used in 441,918 reported suicides in India from 1995 to 2015, 90.3% of which occurred in 11 of the 29 states. There was statistical evidence of lower than expected rates of pesticide suicides (rate ratio [RR] 0.52, 95% CI 0.49–0.54) and total suicides nationally by 2014 (0.90, 0.87–0.93) after the 2011 endosulfan ban. In Kerala, there was a lower than expected rate of pesticide suicides (0.45, 0.42–0.49), but no change to the already decreasing trend in total suicides (1.02, 1.00–1.05) after the 2011 ban of 14 pesticides. The 2005 ban on endosulfan showed a similar effect – lower than expected pesticide suicides (0.79, 0.64–0.99), but no change to the decreasing trend of total suicides (0.97, 0.93–1.02) in 2010. There was no evidence of a decline in agricultural outputs following the bans.

**Conclusion:**

Highly hazardous pesticides continue to be used in India and pesticide suicide remains a serious public health problem. However, some pesticide bans do appear to have impacted previous trends in the rates of both pesticide suicides and all suicides. Comprehensive national bans of highly hazardous pesticides could lead to a reduction in suicides across India, in addition to reduced occupational poisoning, with minimal effects on agricultural yield.

## Background

Self-poisoning with pesticides accounts for 14–20% of global suicides, an estimated 110,000–168,000 deaths each year [1], down from an estimated 371,000 in the late 1990s [2]. The problem is most severe in rural Asian communities, where a wide range of agricultural highly hazardous pesticides (HHPs) are easily available within the home and from shops [3–6]. They are often used impulsively for suicide attempts in times of acute stress [7], frequently with less than 30 min of planning [5]. Surviving an act of pesticide self-poisoning allows people to receive support from their family, community, and medical and psychosocial services, and most suicide attempts are not repeated [8–11].

HHPs have a high case fatality rate in poisoning compared to other agents commonly used for self-poisoning such as analgesics and sedatives [12]. Individuals who do choose another poison, in the absence of HHPs, are likely to choose a less toxic substance that offers a higher chance of survival, after what is often a transient suicidal crisis. This is one example of how means restriction can reduce not only the burden of suicide from that particular method, but also the overall burden of suicide [13–16]. Restriction of access to commonly used, highly lethal suicide methods is widely recognised as one of the most effective suicide prevention strategies [17, 18]. If time and space can be put between a suicidal individual and highly lethal suicide methods, the suicidal impulse may pass, or if they use a less lethal alternative method, their chances of survival are higher [16]. National bans of HHP in several countries have led to large reductions in the number of pesticide suicides and in the total number of suicides where pesticide self-poisoning is a common means of suicide [14].

HHPs of World Health Organization (WHO) toxicity classes Ia, Ib and II - such as the organophosphorus insecticides monocrotophos, phorate, and methyl parathion or the herbicide paraquat [19] - have been responsible for most pesticide suicides worldwide over the last five decades [20, 21]. Pesticide suicide prevention will require a combination of improved medical management, improved community use of pesticides, and government regulation to remove HHPs from agricultural practice [12]. However, of these three interventions, pesticide bans have the most evidence of success internationally [14]. Medical treatment of pesticide poisoned patients is challenging, particularly in remote areas, where patients commonly present late to poorly-resourced hospitals with limited critical care facilities [22, 23]. A large cluster-randomised controlled trial in Sri Lanka has demonstrated that improved HHP storage is unlikely to substantially reduce the number of deaths [24, 25].

Despite a moderate decline in suicide rate over the last 20 years, India still has a large burden of suicide [26]. In women, the rate of suicide is the fourth highest in the world, whilst in men it ranks 62nd [27], corresponding to around 230,000 deaths nationally in 2016. New preventative strategies are therefore greatly needed [28]. Pesticides are frequently used as a method of suicide – the nationally representative Million Death Study estimated that the rate of death by self-poisoning was 7.9 per 100,000 per year for women and 13.8 per 100,000 per year for men, with pesticides used in the majority of these [29]. However, hanging is increasingly common and appears to have offset a decline in pesticide suicides up to 2014 [30]. Multiple observational studies based in healthcare settings have reported pesticide poisoning across India, the vast majority deliberate self-poisonings rather than accidental exposure, with case fatality rates varying from approximately 5% to over 70% [6, 31–41]. Large differences exist between states in the frequency of suicide, with higher rates of suicide recorded in more economically developed states [26, 30, 42] but also states with a higher proportion of the population employed in agriculture [30, 42].

The National Crime Records Bureau (NCRB), a central government body, produces reports on accidental deaths and suicides using data gathered by police forces [43, 44]. It is a useful source of annual data on confirmed cases [30, 44], but local epidemiological studies of the incidence of suicide [3, 45–49] and studies making national estimates from representative samples [26, 29] show that it probably systematically undercounts suicides [44]. In particular, the estimated suicide rates in Bihar and Uttar Pradesh are 19.7 and 8.9 times higher in the Global Burden of Disease study [26] than NCRB rates for 2015 [43].

Pesticide suicide has been relatively neglected as a topic of research in India. Central storage facilities [50] and organic pest management [51] have been tested in small feasibility studies with positive results, but there have been no large-scale intervention trials addressing pesticide suicides specifically.

Pesticides are regulated in India under the Insecticides Act, 1968 [52], and the Insecticide Rules, 1971 [53]. Replacement Pesticide Management Bills proposed in 2008 and 2017 have not yet been passed [54–56]. The Central Insecticide Board (CIB) advises the Ministry of Agriculture and Farmers’ Welfare on pesticide safety [57]. Its mandate includes reviewing matters relating to: (a) the risk to human beings or animals involved in the use of insecticides and the safety measures necessary to prevent such risk, and (b) the manufacture, sale, storage, transport and distribution of insecticides with a view to ensuring safety for human beings or animals [52]. Despite its name, the CIB also advises the government on other pesticides such as herbicides and fungicides. The Registration Committee of the CIB is responsible for deciding which individual pesticide compounds can be registered for production and sale, domestically and for export. The Insecticides Act does not provide for regular review of registered pesticides. Other expert committees occasionally reassess specific registered pesticides if a problem arises, recommending restrictions or bans [58].

The Insecticides Act gives state governments limited powers to regulate pesticides. They may issue licences to companies to manufacture, sell, stock or exhibit for sale or distribute pesticides through application licensing officers. The Act permits states to ban pesticides for 60 days if a safety concern arises, with 30-day extensions in some cases. The states of Punjab, Kerala and Sikkim have developed additional state-level legislation for pesticide regulation beyond the Insecticides Act and have restricted HHP use through this route [59–62].

Several other countries where pesticide suicide is a significant problem have reported on the effects of national pesticide regulation on suicide [14], notably Bangladesh [63], South Korea [64], Sri Lanka [65], but this is the first study, to our knowledge, assessing the effect of national and state-level pesticide regulations on suicide in India. We aim to summarise the pesticide bans and restrictions that have been implemented to date by the central and state governments, and to explore how they might relate to changes in rates of both pesticide suicides and suicides from all methods.

## Methods

Data was collected on the number of pesticides recorded in India, national and state pesticide regulatory actions, and the incidence of suicides nationally and by state from 1995 to 2015. All twenty-nine states were included. Telangana officially separated from Andhra Pradesh in 2014 but was treated as part of Andhra Pradesh for this analysis. The union territories were excluded due to the comparatively small numbers of pesticide suicides (< 0.3% of the total over the 20 years studied) and a lack of official population estimates for 2015 meaning that interpolated suicide rates for the years after the 2011 census could not be calculated. Additional data was collected on agricultural yields over the study period from the Ministry of Statistics and Programme Implementation [66] and on unemployment rate and gross domestic product per capita from the World Bank [67].

### Pesticide toxicity

A list of pesticides registered in India was obtained from the website of the CIB, Ministry of Agriculture & Farmer’s Welfare [68]. The pesticides were then grouped according to the WHO toxicity classification (Ia: extremely hazardous; 1b: highly hazardous; II: moderately hazardous; III: slightly hazardous; and U: unlikely to cause acute hazards) [19]. Of note, the WHO classify pesticides according to their 50% lethal dose (LD50) in mg/kg via both dermal and oral routes in rats as a comparable and reproducible value. This classification does not always translate to case fatality rates in human self-poisoning, with some Class II pesticides such as paraquat and endosulfan having very high case fatality rates after ingestion [69].

### Pesticide regulatory actions

Information on pesticide regulatory actions was obtained for the national level from the CIB website [68]. For regulations at the state level, the official websites of every state’s Agriculture Department were searched using the term “pesticide”, as more specific search terms excluded some relevant documents. Regulatory actions not found through the initial search were identified through media reports and publications from agencies of the United Nations. In these cases, targeted searching with the name of the pesticide and the date of the ban was subsequently used to locate the original government notification pertaining to the ban or restriction where possible. Some compounds had their registration announced but were later omitted from lists of registered pesticides. These omissions were assumed to be errors and the pesticide to be still registered unless there was a government notification specifically announcing a ban on that compound. Permanent bans, temporary bans and partial restrictions were all included but only permanent bans were used in the time series analysis due to concerns about the continuing availability of pesticides under the less strict regulations.

### Pesticide usage

Data on pesticide usage by metric tonne was obtained from the Department of Chemicals and Petrochemicals’ website for financial years 2001/2002 to 2015/2016 [70–72]. This provided usage data for several individual compounds at the national level, and data for overall pesticide usage at the state level. However, data on usage of individual compounds at the state level was not available so we were unable to assess if state-wide bans reduced usage within that state. Pesticide use was estimated by the Department of Chemicals and Petrochemicals by subtracting the quantity exported and adding the quantity imported to the quantity produced domestically. This methodology does not adjust for differences in stockpiling from year-to-year or any inaccuracies in reports from importing or manufacturing firms.

### Suicides

Suicide data were extracted from the NCRB’s annual reports for the years 1981 to 2015 [43]. A suicide is defined by the NCRB as an unnatural and deliberate termination of life, when the desire to die originates within the individual and there is a reason for ending that life. Methods used in recorded suicides are classified into 12 categories: insecticides, other poisons, drowning, self-immolation, firearms, hanging, overdose of sleeping pills, self-inflicting injury, jumping (from height or from moving vehicles/trains), being hit by vehicles/trains, touching electric wire, and other means. It is unclear whether the insecticide class includes all forms of agricultural pesticides, including herbicides, as the document also refers to “insecticides/pesticides”; we assumed that deaths recorded as insecticide self-poisoning also included other pesticides. The NCRB statistical reports do not provide detail on how the data on means of suicide are gathered. Prior to 1995, pesticides did not have their own category as a means of suicide, being included in a ‘poisons’ category. Data for all forms of poisoning (including pesticide) suicides were therefore extracted for 1981–2015 to identify longer-term trends.

Suicide rates were calculated using census population records for the years 2001 and 2011, and using official estimates for each state for 2015 [73–75]. Official state-wise population records from the 1991 census were implausibly low and did not correspond to national population records, so were not used. Official population estimates for 2015 were only available for the 20 largest states. Populations for intervening years were estimated using interpolation. Suicides in NCRB records were not stratified by age for each individual state, so crude mortality rates were used. Both pesticide suicides and total suicides (including all methods of suicide) were included. Maps displaying data by state were created using mapchart.net under a Creative Commons Attribution-Share Alike 4.0 International licence [76]. This study did not investigate gender as a factor influencing suicide rates, although previous studies using the NCRB and other sources have noted that suicide rates amongst Indian women have fallen by 22–26% over the past 20 years, while rates in Indian males have remained stable [26, 77]. We combined data on male and female suicides, as we have no reason to believe pesticide regulation will affect males any differently than females - this method is commonly used by both sexes. Furthermore, in view of the limited number of data points (years) included in this study, we did not have the statistical power required to undertake multivariable analysis.

### Statistical analysis

We used Joinpoint regression analysis [78] to investigate trends in suicide rates between 1995 and 2014, i.e. all years when pesticide suicide data were available apart from the possibly artefactual rise in pesticide suicide seen in some states in 2015. Joinpoint regression identifies time points (years) when the trend has changed from a stable trend (join points) and includes them in the model if the change is significantly different from zero at the alpha = 0.05 level. This does not involve an a-priori assumption of when an intervention thought to affect the outcome occurred and is useful in identifying an appropriate time period of stable pre-intervention trends to use in interrupted time series analysis.

**Fig. 1:**
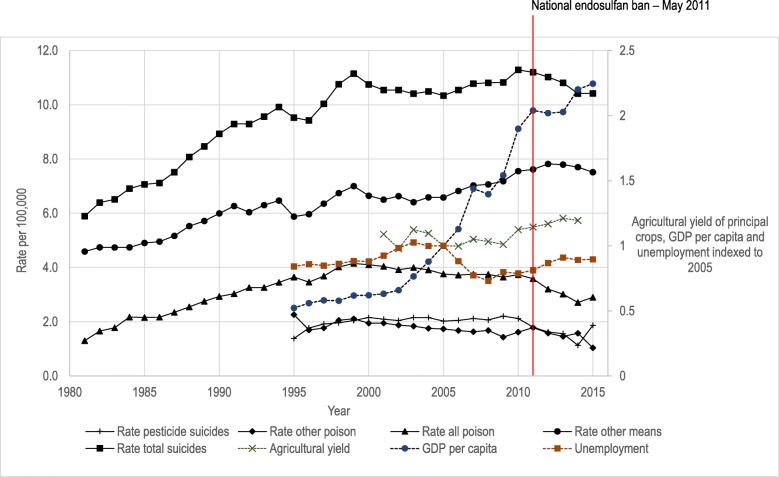
Annual incidence per 100,000 population of ‘total suicides’, ‘pesticide suicides’, ‘other poisoning suicides’ and ‘suicides by other means’ from 1995 to 2015, with annual yield of principal crops from 2001 to 2014, unemployment, and GDP per capita centred on 2005

A-priori, based on our review of state and national pesticide regulations (Table 1) where the date of the ban was recorded, we identified three relevant bans to investigate: the endosulfan bans in Kerala (2005) and all India (2011), and Kerala’s ban of 14 pesticides in 2011. The ban of aldicarb in 2001 was not investigated, as reports of it being used for suicide in India are very rare [21, 79]. We carried out an interrupted time series analysis for these three periods, analysing trends in suicides by all methods and pesticide suicides, using the periods of stable pre-ban trends identified using joinpoint regression. There was statistical evidence of over-dispersion in the Poisson regression models, and therefore we used negative binomial regression to compare suicide rates after these bans with those predicted based on pre-ban trends. We calculated rate ratios for each year after each ban compared with predicted rates based on extrapolated trends before each ban. 2015 was excluded from our primary analysis due to a sudden large increase in rate of pesticide suicide from 2014. The synchronous fall in suicides by “other poisons” and rise in pesticide suicides (Fig. 1) suggests that the sudden increase was artefactual. A sensitivity analysis was also conducted including the year 2015 to check if this assumption changed our conclusions. Stata version 15 [80] was used for the regression analysis.

**Table 1:** Timeline of national and state pesticide bans

Date	Territory	Pesticide banned	State bans
**1974**	National	**parathion (ethyl parathion**)	
**1989**	National	dibromochloropropane, pentachloronitrobenzene, toxaphene	
**1990**	National	**endrin**	
**1996**	National	aldrin, chlordane, heptachlor	
**2001**	National	aldicarb, chlorbenzilate, dieldrin, ethylene dibromide, maleic hydrazide, trichloroacetic acid	
**2005**	National	(dalapon, ferbam, formothion, nickel chloride, paradichlorobenzene, simazine, warfarin)	
**2005**	Kerala		DDT, **endosulfan**
**Before 2007**^**a**^	National	benzene hexachloride, calcium cyanide, copper acetoarsenite, ethyl mercury chloride, menazon, nitrofen, **paraquat dimethyl sulphate**, pentachlorophenol, phenyl mercury acetate, sodium methane arsonate, tetradifon	
**2011**	Kerala		anilofos, atrazine, **carbofuran**, edifenphos, methoxy ethyl mercuric chloride, **methyl parathion**, **monocrotophos**, oxythioquinox, **paraquat**, **phorate**, **profenofos**, thiobencarb, triazophos, tricyclazole
**2011**	Karnataka		[**endosulfan**]
**2011**	National	**endosulfan**	
**2007 to 2012**^**a**^	National	**chlorofenviphos**, metoxuron	
**2013**	National	lindane	
**2014**	Sikkim		all pesticides
**2014**	National	(sirmate)	
**2017**	Maharashtra		[**acephate**, **cypermethrin**, diafenthiuron, fipronil, imidacloprid, **monocrotophos**, **profenofos**]
**2018**	Punjab		alachlor, benfuracarb, bifenthrin, **carbosulfan**, chlorfenapyr, dazomet, dicofol, diflubenzuron, **endosulfan**, ethofenprox, **fenitrothion**, kasugamycin, metaldehyde, **methomyl**, **monocrotophos**, **phorate**, **phosphamidon**, thiophanate-methyl, triazophos, tricholorofon
**2018**	National	benomyl, **carbaryl**, **diazinon**, fenarimol, **fenthion**, linuron, methoxy ethyl mercuric chloride, **methyl parathion**, thiometon, tridemorph	
*2020*	*National*	*alachlor,* ***dichlorvos****,* ***phorate****,* ***phosphamidon****, triazophos, trichlorfon*	

**Key:** HHPs frequently used for suicide are indicated in **bold**. Pesticides withdrawn from use until further information as requested by the Registration Committee is submitted are in (parentheses). Temporarily banned pesticides are in [square brackets]. Proposed future bans on pesticides are in *italics*

^**a**^information on which year these pesticides were banned is not available

## Results

Our search identified a total of 26 documents relating to pesticide regulations and bans: four documents from state governments [60, 62, 81, 82] and 12 from the central government [83–94], five media reports relating to state regulations [54, 61, 95–97], three media reports relating to national regulations, [98–100] and two documents published by agencies of the United Nations [101, 102]. We found no documents related to any state-wide pesticide bans for 24 of the 29 states over the period studied.

As of October 2019, 318 pesticides were registered in India, twelve with some restrictions on their use (supplementary table) [83, 84]. Four of these pesticides are WHO toxicity class Ia (extremely hazardous) compounds (bromadiolone, captafol, phorate, phosphamidon) while fourteen are WHO toxicity class Ib (highly hazardous) compounds (beta-cyfluthrin, carbofuran, coumatetralyl, cyfluthrin, dichlorvos, edifenphos, methomyl, monocrotophos, oxydemeton-methyl, propetamphos, sodium cyanide, tefluthrin, triazophos and zinc phosphide). Many of these compounds are used within India at rates of several thousand tonnes annually [70], indicating widespread availability of pesticides with high acute toxicity.

There are also 95 registered pesticides of WHO class II hazard, some of which are highly toxic after ingestion [22], with case fatalities often greater than 10% as shown by a large prospective secondary hospital case series from Sri Lanka [69] (paraquat 42.7%, dimethoate 20.6%, quinalphos 12.1%, alachlor 11.1%, profenofos 11.0%, propanil 10.9%, and carbosulfan 10.7%) [69]. Thirty-three class III (slightly hazardous) pesticides and 48 class U (unlikely to present acute hazard) pesticides are registered.

Three fumigants are registered: aluminium phosphide, DD mixture (dichloropropene and dichloropropane), and methyl bromide. Although not classified by the WHO, aluminium phosphide is extremely toxic after self-poisoning, with a case fatality often exceeding 50% after ingestion of the previously common 56% 3 g tablets [31, 33, 36–38, 41].

An additional 117 non-fumigant pesticides registered for use in India are not yet classified by the WHO for toxicity, and five pesticides listed by the WHO as obsolete are also registered for use. None of these compounds have their consumption reported by the Department of Chemicals and Petrochemicals [70], which could indicate that they do not constitute a large part of the market. 36 pesticides included on earlier lists of registered pesticides were omitted from more recent documents without any ban or withdrawal being announced – only two (nicotine sulfate and tefluthrin) are highly hazardous, and neither of them have been reported as common methods of suicide.

### National regulatory actions

Since 1989, 39 pesticides have been banned nationally (Table 1) [85, 86, 98, 101, 103], including ten HHPs (bold in Table 1) identified in previous studies as being important for suicide in South Asia [22, 32, 34, 39, 40, 69, 104]. An additional 26 pesticides have been refused registration (supplementary table, footnote) or withdrawn from the market (Table 1, footnote) [83, 85, 87–90]. Only bans that covered hazardous and commonly used (according to the Department of Chemicals) pesticides were further analysed by joinpoint regression.

In 2015, the Indian government set up the Anupam Verma Committee to review the continued use of 66 pesticides that have been banned or restricted for farming use in other countries [58]. In 2016, it recommended a ban on 13 pesticides, phasing out of 6 pesticides by 2020, and further review of 27 pesticides in 2018 [91, 92]**.** The Ministry of Agriculture partially implemented the recommendations in August 2018, banning 10 pesticides, placing restrictions on 2, and scheduling six bans for 2020 including several WHO Class Ia HHPs (Table 1). Two pesticides had been recommended for a complete ban but were only restricted: sodium cyanide and trifluralin. DDT (dichlorodiphenyltrichloroethane) was not banned and its sole permitted use by the Ministry of Health was maintained.

Endosulfan was banned by the Supreme Court of India in May 2011, with the final stocks disposed of or exported by January 2017 [85]. According to the Department of Chemicals and Petrochemicals, no more endosulfan was produced domestically after the ban. As might be expected, an increase in the use of pesticides which have the same applications as endosulfan was also reported [70], including the WHO class II organophosphate insecticides profenofos, chlorpyriphos, and acephate.

Overall, these regulatory actions have included national bans of ten HHPs that are relevant to pesticide suicides (Table 1). Another eleven have been restricted in their use, for example ‘not to be used on vegetables’, or are only available in certain formulations (supplementary table). However, effective enforcement of these partial restrictions has proven difficult [93, 99, 100]. Monocrotophos, for example, despite being banned for use on food crops, is still widely used by farmers on vegetables as well as on its main permitted use for cotton [93, 102], as demonstrated by the Ministry of Agriculture’s “Monitoring of Pesticide Residues at National Level” scheme frequently identifying monocrotophos at above the maximum residue limit in samples of vegetables from markets and at the farm gate [92, 94].

### State regulatory actions

Kerala, Punjab and Sikkim have passed separate laws permanently banning some pesticides, whilst Karnataka and Maharashtra have implemented temporary bans.

Kerala permanently banned endosulfan in October 2005 [82] and 14 other pesticides, many relevant for suicide, in January 2011 – two WHO class Ia, four class Ib, five class II, two class III and one listed by the WHO as obsolete (Table 1) [59, 81]. Bans for some of these pesticides have now been announced by the Central Government: methoxy ethyl mercuric chloride and methyl parathion in 2018 and phorate and triazophos in 2020. However, nine pesticides banned in Kerala remain in use nationally with no plans for regulatory action (anilofos, atrazine, carbofuran, edifenphos, monocrotophos, paraquat dichloride, profenofos, thiobencarb and tricyclazole).

Punjab, using the provision of the Insecticides Act that allows states to refuse renewal of pesticide licenses once they expire, decided not to renew licenses of 20 pesticides in 2018, including the HHPs carbosulfan, endosulfan, fenitrothion, methomyl, monocrotophos, phorate and phosphamidon (Table 1) [54, 60, 95]. Sikkim banned all inorganic agricultural inputs, including HHP, in 2014 under the Sikkim Agricultural, Horticultural Input and Livestock Feed Regulatory Act [62]. Pesticides were withdrawn from agricultural use in the state by 2016 [61].

Temporary bans have taken place in Maharashtra and Karnataka (Table 1). In November 2017, Maharashtra state requested that the Central Government ban five pesticides inhaled by victims of an accidental mass poisoning in Yavatmal district. The state also banned five formulations of these compounds for 60 days, including acephate 75% and monocrotophos 36% (Table 1). The ban only applied to five districts and other formulations were still permitted [96]. Karnataka banned endosulfan in February 2011 for 60 days [97], shortly before the Supreme Court banned the compound nationally in May of that year [85]. Kerala was thus the only state that applied permanent pesticide bans within the period studied, and was, therefore, the only state for which we performed joinpoint regression to assess the effects of those bans.

### Suicides

The NCRB recorded 133,623 deaths from suicide in 2015 [43], of which 23,930 (17.9%) were due to pesticides. From 1995 to 2015, there were 2,451,410 suicides from all methods and 441,918 pesticide suicides (18.0% of the total) recorded in India. Suicide rates from all methods, all poisons, pesticides, other poisons, and all other methods are shown in Fig. 1. After rising steadily to 1999, the total suicide rate as reported by the NCRB remained relatively stable until 2011, at which point it began to decline. The rate of pesticide suicides rose sharply in 2015, accompanied by a corresponding decline in suicides from other poisons.

Also presented in Fig. 1 is the combined national agricultural yield in kg/hectare of rice, wheat, cotton and 26 other important crops, as compiled by the Ministry of Statistics and Programme Implementation. This data is indexed to the yield recorded in the year 2005 and shows an increasing trend despite the pesticide bans that have taken place. Economic growth averaged 7.85% per year over the study period, with the only major recession occurring at the time of the global financial crisis in 2008. Unemployment was relatively stable at around 2.7%, with an increase to 3.2% in 2003 before falling to 2.3% in 2008, then increasing back to around 2.7% by 2015. Changes in both these factors were not suggestive of any effect on suicides (Fig. 1).

The majority of pesticide suicides (90.3%) occurred in eleven of the 29 states: Maharashtra, Andhra Pradesh, Madhya Pradesh, Tamil Nadu, West Bengal, Kerala, Telangana, Karnataka, Gujarat, Odisha and Chhattisgarh**.** These states account for approximately 54.1% of the total population of India [74], and 84.2% of suicides by all methods in India. Supplementary figure 1 shows the annual absolute number of pesticide suicides from each state. Supplementary figure 2 shows the sum of the same data from 1995 to 2015 in map format. Maharashtra had the largest total number of pesticide suicide deaths from 1995 to 2015 with 84,194 (19.2% of total), followed by Andhra Pradesh with 77,394 (17.6% of total).

**Fig. 2:**
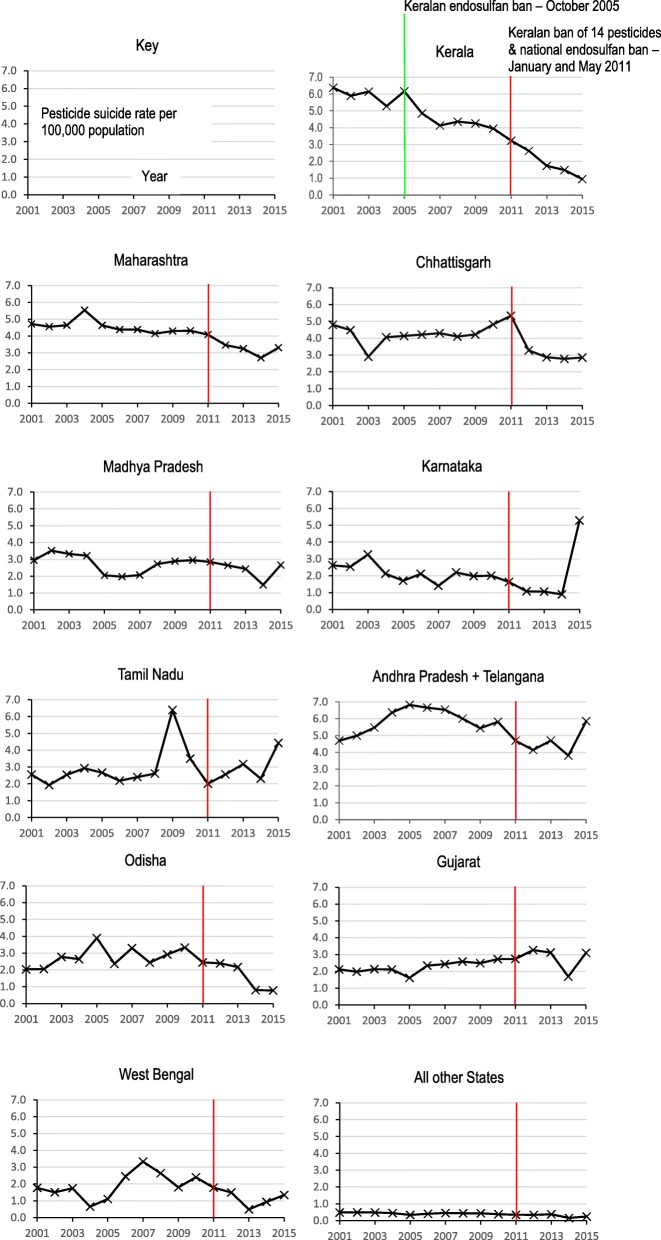
Incidence of pesticide suicide by state from 2001 to 2015

**Fig. 3:**
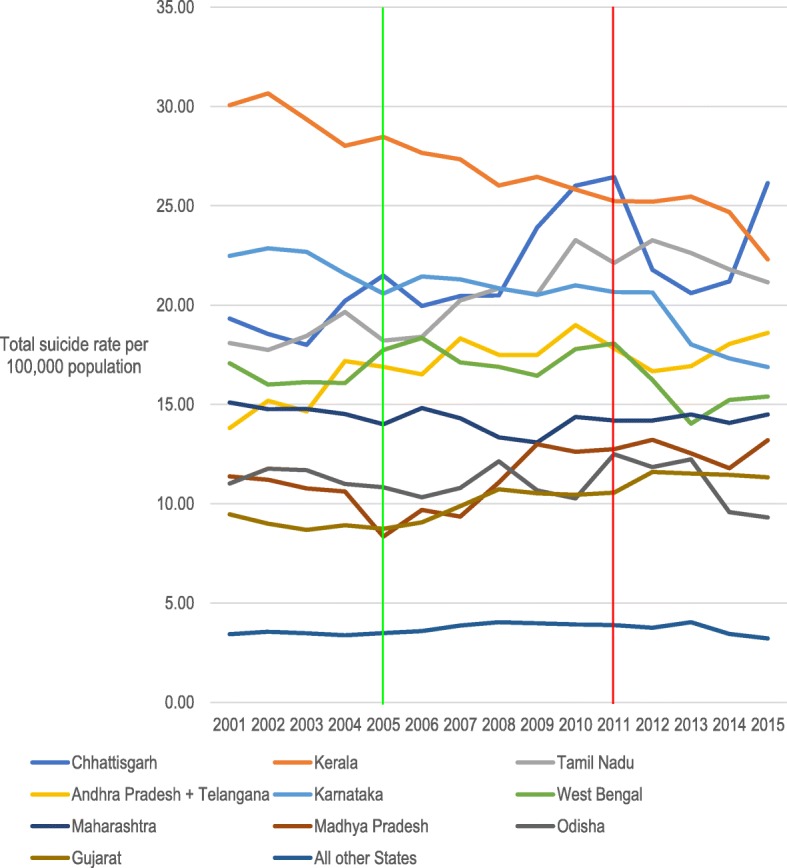
Incidence of all suicides by state from 2001 to 2015

Throughout most of the study period the pesticide suicide rate was highest in Andhra Pradesh and Telangana. Annual pesticide suicide rates for the eleven states with the highest numbers of pesticide suicides are plotted in Fig. 2 and the change in pesticide suicide rates for all states in map format in supplementary figure 3. Equivalent data for suicides by all methods is displayed in Fig. 3 and supplementary figure 4, where Kerala had the highest rate for most of the study period before being superseded by Chhattisgarh.

The national total and pesticide suicide rates were lower than expected, based on previous trends, for each year after the 2011 national ban on endosulfan (Table 2). The reduction was larger for pesticide suicide (48% [95% CI 46 to 51%] lower than expected by 2014) than total suicides (10% [95% CI 7 to 13%] lower than expected by 2014).

**Table 2:** Rate ratios for overall and pesticide suicide rates after the 2011 ban of endosulfan (throughout India) and 14 pesticides (Kerala)

	Rate ratios (95% CI)			
	National suicides		Kerala suicides^c^	
	Total^a^	Pesticide^b^	Total	Pesticide
Post-ban years				
2011	1.00 (0.98, 1.02)	0.83 (0.81, 0.86)	0.99 (0.98, 1.01)	0.85 (0.80, 0.91)
2012	0.97 (0.95, 1.00)	0.75 (0.72, 0.77)	1.01 (0.99, 1.03)	0.73 (0.68, 0.78)
2013	0.95 (0.92, 0.97)	0.71 (0.69, 0.74)	1.04 (1.01, 1.06)	0.51 (0.47, 0.55)
2014	0.90 (0.87, 0.93)	0.52 (0.49, 0.54)	1.02 (1.00, 1.05)	0.45 (0.42, 0.49)

Period of consistent linear trend prior to ban: ^a^2003–2010; ^b^1997–2010; ^c^1999–2010

In Kerala, after the 2011 ban on 14 other pesticides, the rate of pesticide suicides fell further than expected based on previous trends (55% [95% CI 51–58%] lower than expected in 2014), but there was no evidence of a change to the pre-existing downward trend in total suicides, unlike the change in national suicides (Table 2). The 2005 Keralan ban on endosulfan similarly did not appear to affect the trend in total suicides, but there was statistical evidence of a reduction in pesticide suicides rates compared to pre-ban trends (1999–2005) (Table 3).

**Table 3:** Rate ratios for overall and pesticide suicide rates after the 2005 endosulfan ban in Kerala

	Kerala suicides*	
	Total	Pesticide
Post-ban years		
2006	0.99 (0.96, 1.02)	0.87 (0.77, 0.99)
2007	0.99 (0.96, 1.02)	0.77 (0.66, 0.89)
2008	0.95 (0.92, 0.99)	0.83 (0.70, 0.99)
2008	0.98 (0.94, 1.03)	0.83 (0.68, 1.01)
2010	0.97 (0.93, 1.02)	0.79 (0.64, 0.99)

*Period of consistent linear trend prior to endosulfan ban 1999–2005

The large increase in pesticide suicides in 2015 (Fig. 1) was mostly due to increased numbers in Karnataka (increase of 2818, or 501.4%), Tamil Nadu (increase of 1591, or 92.9%) and Andhra Pradesh (increase of 1830, or 55.1%) (supplementary figure 1). However, all three of these states saw large decreases in suicides coded as “consuming other poison” over the same period – 2138 for Karnataka (− 98.6%), 1142 for Tamil Nadu (− 30.4%) and 1168 for Andhra Pradesh (− 72.0%). These are nearly as large as the increases in pesticide suicides, suggesting that changes in coding may have contributed to the rise. Inclusion of the year 2015 in our time series analysis changed one of our conclusions – there was evidence of a decline of total suicide rate as well as pesticide suicide rate in Kerala during that year relative to 2011. The increase in the national pesticide suicide rate in that year was not large enough to change our conclusions about the trend since 2011 (Table 3).

## Discussion

According to Indian police data, over 20,000 Indians died in 2015 from pesticide self-poisoning. After a steady rise in suicides from 1981, there have only been relatively small changes in the overall suicide or pesticide suicide rates nationally since 2001. This stands in contrast to neighbouring Sri Lanka and Bangladesh, where both total and pesticide suicide rates have fallen dramatically after pesticide regulation removed most HHPs from national agricultural practice [63, 105]. Our analysis does suggest an impact of pesticide restrictions in India – the 2011 national endosulfan ban was associated with a small but significant decrease in total suicide rates and a larger decline in pesticide suicide rates. However, since many other highly hazardous pesticides remained available, switching to another highly lethal means of suicide was easy. WHO class I pesticides were still widely available, and the usage of other class II pesticides increased [70, 71], although the class II pesticides with similar uses to endosulfan which seem to have replaced it (acephate, profenofos and chlorpyriphos) do have somewhat lower case fatality rates in poisoning (0, 11 and 7.6% respectively [69], compared to 22–30% for endosulfan [40, 69, 106]). The overall impact of this ban is likely to have been attenuated compared to the effects of more widespread restrictions seen in other countries [63, 64, 105].

The marked fall in total suicides in Sri Lanka followed removal of all Class I pesticides from agriculture. This left only comparatively lower toxicity pesticides accessible to people in a suicidal crisis. The incidence of non-fatal self-poisoning actually increased [107], but the use of less lethal pesticides caused the national total suicide rate to decline [65]. Additionally, most people who survive a first attempt do not repeat their act [8, 11]. A similar effect could potentially be seen in India, especially with the broader bans that are taking place in 2018 and 2020. Whether these bans constitute enough of a reduction in access to lethal means to have a significant effect should be assessed in future research.

The fall in total suicide deaths for all India noted from 2011 to 2014 (3919) was smaller than the fall in pesticide suicide deaths (7463). This suggests that there was some means substitution occurring, but not enough to negate the large drop in pesticide suicides.

Both pesticide suicides and total suicides were already falling in Kerala by the time the 2005 endosulfan ban and the 2011 ban of 14 pesticides were implemented. Our analysis does suggest an acceleration in the rate of decline in pesticide suicides after the 2011 ban, but there was no evidence of impact on the overall rate of suicide. One possible contributory factor to the decline in pesticide suicide in Kerala is the state’s comparatively rapid urbanisation [74, 108] leading to fewer households having direct access to agricultural pesticides even before the bans were implemented, an effect also seen in Taiwan [109]. As with the national trend, there was also some means substitution, attenuating the fall in overall suicides.

The year 2015 saw a concerning increase in recorded pesticide suicides in nearly all Indian states, with the largest increases in Karnataka, Tamil Nadu, and Andhra Pradesh. This may explain the contrast in our conclusions with other publications using NCRB data that have reported reductions in the incidence of all poisoning suicides from 2001 to 2010 [42] and of pesticide suicides from 2010 to 2014 [30]. The increase in 2015 might reflect differences in reporting rates or coding accuracy, but the influence of these factors is unclear without publicly available methodology for the NCRB reports from each year. However, when compared with the higher rates of pesticide suicide and suicide from all methods reported by more rigorous epidemiological studies [26, 29], it seems likely that the increase brings the NCRB rate closer to reality. There was also a synchronous decline in self-poisoning using substances other than pesticides in that year, particularly in the states with the largest increases in pesticide poisoning, as noted above. The overall decline in poisoning suicides (Fig. 1) suggests that pesticide bans are effective regardless of changes in coding. Nevertheless, further research investigating later years is necessary to clarify whether the increase is real and sustained. If 2015 is included in our time series analysis, one key conclusion is altered – there is evidence of a lower than expected rate of total suicides as well as pesticide suicides in Kerala after the 2011 bans, but the delay casts some doubt on whether the pesticide regulations were the most important factor in this.

Although there has been regulatory activity in India over the last 20 years at both national and state level, HHPs continue to be widely used in agriculture and used in tens of thousands of suicides each year. In August 2018, a few key HHPs were banned nationally; a further six including three important HHPs often used in suicide (dichlorvos, phorate and phosphamidon) are scheduled for bans in 2020. If they are to be more effective in reducing suicides, new regulations will need to be properly enforced – there have previously been reports of smuggling of banned pesticides across state borders [110, 111] which would reduce the effectiveness of the bans. This problem would, however, be insignificant for national bans, particularly with chemicals such as monocrotophos which are not manufactured in significant quantities outside of India. There is also a risk that future bans could be circumvented by manufacturers within the country. Inspections, and strict sanctions for firms failing to comply, are likely to be needed, together with ensuring that cost-effective and safe alternatives are widely available to farmers.

Sikkim has banned all pesticides from agricultural use, but the ban only came into effect in 2016, a year after the data available for this analysis. The effects of the fairly extensive 2018 bans in Punjab and nationally will likewise require further research.

The number of suicides reported by the NCRB is likely to be a substantial underestimate of the actual number of suicides in India due to under-reporting [46, 112, 113]. The legal status of attempted suicide remains ambiguous. It is a crime according to Section 309 of the Indian Penal Code (IPC) [114]. The Mental Healthcare Act 2017 [115] decriminalized suicide, affirming a “presumption of severe stress in cases of attempt to commit suicide”, but the IPC was not amended and Section 309 remains in place. Significant social stigma also continues to surround the issue of suicide in India [116], which is also likely to reduce reporting.

There is currently no specific national suicide prevention strategy in India, which many, including the WHO, have called for [117, 118], but reducing access to highly hazardous pesticides should be considered in further efforts to prevent suicide [119]. Some specific HHPs with no bans currently scheduled stand out as being of highest priority for future bans [119]. All WHO class Ia and Ib pesticides are frequently lethal in self-poisoning and occupational poisoning, and should have no place in routine agricultural practice in small-scale farms without the resources to store or use them safely [120]. Monocrotophos (class Ib) has been highlighted by the WHO as being particularly damaging to India’s health [102] and, as one of the most widely used pesticides [70, 93, 121], a total ban could have a large effect in reducing access to the means of suicide. Monocrotophos also illustrates the problem of just restricting pesticides to certain uses - it is banned for use on vegetables [85] to protect consumers from residues in their food [122], but its widespread use in cotton production means it is still easily available in shops for illegal use in vegetable production. Other pesticides with high case fatality rates in self-poisoning for which bans should be considered include WHO toxicity class II HHPs paraquat, profenofos, quinalphos, dimethoate, and carbosulfan, as well as the extremely toxic fumigant aluminium phosphide, which is still used in a large number of self-poisoning deaths, primarily in the north of the country [31, 33, 36–38, 41]. A promising change to aluminium phosphide regulation was noted in a 2008 paper from Chandigarh, where case fatality ratios for acute poisoning from the compound dropped after 2000, possibly due to restrictions on the sale of tablets of aluminium in favour of loose powder sachets [35].

An argument often given in favour of limiting restrictions on pesticides is that inexpensive pesticides are necessary to maintain agricultural productivity [123, 124]. However, this claim does not specifically apply to HHPs, as integrated pest management (IPM) and less hazardous but still inexpensive pesticides are available as effective alternatives [125, 126]. Yields increased over the time period of this study despite the pesticide bans that have taken place (Fig. 1). In other Asian countries that have banned some or all HHPs, such as Sri Lanka, Bangladesh, and South Korea, no effect on agricultural output has been seen [15, 63–65, 125]. The economic status of farmers and their families is also negatively affected by HHPs - occupational exposure and self-poisoning lead to expensive medical bills even for those who survive [23], as well as reduced family income and increased debt burden due to death or disability [127].

Since the passing of the Insecticides Act 60 years ago, understanding of pesticide management and the harms associated with their use has improved, and international guidance has changed [119, 128]. The Act does not currently enable state governments to ban pesticides long-term. The 2017 Draft Bill aimed to extend the duration of a state ban from 90 to 240 days [56]. However, to address the harm done by pesticides on their territories, states should probably be able to permanently ban pesticides that are locally problematic. Temporary bans seem to have little effect on the availability of HHPs for agricultural use or for suicides, as normal use and sale is reinstated once the ban is over and there is no provision within the Insecticides Act for the recall of existing stocks [52]. Pesticide registration could be reviewed regularly, with registration routinely valid for perhaps 5 years. A more precautionary approach to registering new pesticides, taking into account the likely toxicity in self-poisoning in addition to that from inadvertent exposure, would reduce the chances of banned pesticides being replaced by similarly lethal new pesticides. Effective enforcement of regulations is also needed. The actions of the government of Kerala are an example to other regional governments in Asia. Its Agricultural Development Policy acknowledges the harms inflicted on farmers and society by the use of HHPs. A key objective of the policy is to minimise the use of HHPs by ensuring that farmers can access chemicals of biological origin, reducing the quantities of pesticides used, and imposing continuous restrictions on the use of HHP [129]. Sikkim has been recognised by the United Nations for being a world leader in organic agricultural production, banning all pesticides [61], although data analysed in this study did not extend to 2016, so we could not assess the effects of this ban.

The Standing Committee on Agriculture, in a report to the Lok Sabha (the lower house of India’s Parliament) has acknowledged that excessive use of pesticides has led to high levels of pesticide residues in food and animal feed, accumulation of dangerous persistent organic pollutants, possible increased rates of cancer, increased input costs of agriculture [130] and farmers suffering a wide variety of adverse health effects from occupational exposure to pesticides [131]. However, relatively little attention has been focused on the link between HHPs and suicide in India. Other countries have demonstrated that pesticide regulation is probably the most effective approach to suicide reduction in places where pesticides are an important means of suicide [14]. HHP bans may also result in marked reductions in the incidence of occupational and unintentional pesticide poisoning [132, 133]. Additionally, banning all HHPs could support India’s efforts to meet, among others, target 3.4 of the United Nations’ Sustainable Development Goals [134] - to reduce by one third premature mortality from non-communicable diseases including suicide.

### Limitations

This quasi-experimental study can provide an estimate of the effect of bans but cannot confirm a causal link between pesticide regulations and suicide. It is possible that other factors have influenced the recent trends. However, we decided to perform an unadjusted time series analysis for several reasons. The period studied only includes four data points post-intervention – the 4 years after the 2011 bans. This means that we cannot assess lag effects and, importantly, that the statistical power required to perform multivariable analysis is lacking.

Variables such as age, religion and gender distribution change slowly, unlike a pesticide ban which should take effect relatively rapidly, leading to the step change in the trend of suicide rate seen in this data. Additionally, pesticide self-poisoning is a common method of suicide for both males and females and for every age group, so bans would likely have an effect on the whole population.

Bans might affect rural or urban areas differently as seen in Taiwan [109], and suicide rates could be affected by increasing rural to urban migration [135], but this is a topic for further research. The NCRB doesn’t report if suicides were in a rural or urban area, only which state they occurred in. A small proportion (14% in 2015) of recorded suicides took place in a selected group of 53 cities which are reported separately to state-wise suicides, but this data is not sufficient to determine how bans affect rural or urban areas generally, as only the largest cities are included.

More rapidly changing factors such as unemployment and economic growth can also affect the overall suicide rate [135]. However, such factors would likely affect both pesticide suicides and suicides using other methods – and this does not appear to be the case (Fig. 1) where trends in poisoning and other methods of suicide diverge. National changes in unemployment and GDP per capita did not appear to be related to either total suicides or pesticide suicides over the time period studied here (Fig. 1).

Bias that differs between states in the likelihood of suicides being recorded probably has a large effect. This is particularly shown by Bihar and Uttar Pradesh, where suicide rates reported in the more rigorous Global Burden of Disease study were 19.7 and 8.9 times higher respectively [26] than those reported by the NCRB, as mentioned previously. This undermines the predictive value of controlling for known variables as an unreliable estimate could still be made.

In addition to our unadjusted analysis, use of the NCRB reports as a source of data lead to several other limitations in this study, including: minimal description of how the data is gathered each year (so reporting of suicides may have changed over time, perhaps explaining the increase in 2015); likely underreporting due to the data being gathered by police officers in the context of what was until recently an illegal act; potentially large amounts of misclassification (> 20% of suicides coded as “other poison” or “other means”) and no post-mortem laboratory confirmation of the poisoning agent used. Monthly data on suicides would enable improved accuracy of time series analysis. The NCRB reports for 2017 and 2018 have not yet been released.

A recent paper by Arya and colleagues [30] also uses the NCRB data and corrects for confounding factors, but analysed states in groups based on socio-demographic factors rather than regulatory status which often only affects one state. The methodology used in Patel and colleagues’ paper [29] gives a more accurate point estimate of mortality rates from suicide, but does not show the change in rate over time in response to changing regulations. Further research would ideally use individual level data to generate suicide death rates, including the specific poison or other method used, from representative samples in each state. These could be followed up over time to assess for any changes in response to further regulations, and adjustments made for confounding variables.

An additional weakness of our study is that it has not assessed the effects of bans announced after 2015. Suicide data from 2016 and later will need to be reviewed to consider the effectiveness of further pesticide regulations and measures to reduce the burden of suicide in India. Bans in Sri Lanka typically demonstrated initial effects within 2 years [14, 136].

Finally, our literature search for notifications of bans had some limitations. The nature of the various state and central government websites where the notifications are stored made systematic searching challenging, so a more opportunistic search strategy was necessary which may have omitted some regulations. Although English is an official language in India, most central government documents we used were also published in Hindi. One notification from Kerala was written in Malayalam [59], with only the names of pesticides being the same in English, although this ban was cross-referenced with another document from that state’s government describing the ban in English [81]. It is thus also possible that some documents concerning pesticide bans written in Hindi or other official state languages have been missed.

## Conclusions

This study suggests that pesticide regulation in India may have had an effect on the total suicide rate nationally and the pesticide suicide rate in Kerala, corroborating the effect demonstrated by pesticide bans in other South Asian countries where pesticide self-poisoning has been a common method of suicide. Further research is required to assess the effects of restrictions after 2015, and better-quality data including further representative samples will be beneficial in assessing the effect of this and other interventions to reduce suicide. However, it is clear that HHP bans should be considered as part of a broader national suicide prevention strategy in India.

## Supplementary information

**Additional file 1: Table S1.** Timeline of partial restrictions. **Figure S1.** Annual pesticide suicides by state from 1995 to 2015. **Figure S2.** Map of total number of pesticide suicides by state from 1995 to 2015. **Figure S3.** Map of change in rate of pesticide suicide by state from 2001 to 2015. **Figure S4.** Map of change in rate of total suicide by state from 2001 to 2015.

## Data Availability

All data are available from public websites as reported in the paper.

## Abbreviations

CIB: Central Insecticides Board
DDT: Dichlorodiphenyltrichloroethane
HHP: Highly hazardous pesticide
IPC: Indian Penal Code
LD50: Median lethal dose (dose required to kill half the members of a tested population)
NCRB: National Crime Records Bureau
WHO: World Health Organization

## References

[1] Mew EJ, Padmanathan P, Konradsen F, Eddleston M, Sen CS, Phillips MR (2017). The global burden of fatal self-poisoning with pesticides 2006–15: Systematic review. J Affect Disord.

[2] Gunnell D, Eddleston M, Phillips MR, Konradsen F (2007). The global distribution of fatal pesticide self-poisoning: systematic review. BMC Public Health.

[3] Bose A, Sandal Sejbaek C, Suganthy P, Raghava V, Alex R, Muliyil J (2009). Self-harm and self-poisoning in southern India: choice of poisoning agents and treatment. Trop Med Int Heal.

[4] Weerasinghe M, Pearson M, Peiris R, Dawson AH, Eddleston M, Jayamanne S (2014). The role of private pesticide vendors in preventing access to pesticides for self-poisoning in rural Sri Lanka. Inj Prev.

[5] Eddleston M, Karunaratne A, Weerakoon M (2007). Choice of poison for intentional self-poisoning in rural Sri Lanka. Clin Toxicol.

[6] Chowdhury AN, Banerjee S, Brahma A, Weiss MG (2007). Pesticide practices and suicide among farmers of the Sundarban region in India. Food Nutr Bull.

[7] Phillips MR, Yang G, Zhang Y, Wang L, Ji H, Zhou M (2002). Risk factors for suicide in China: a national case-control psychological autopsy study. Lancet.

[8] Carroll R, Metcalfe C, Gunnell D (2014). Hospital presenting self-harm and risk of fatal and non- fatal repetition: systematic review and meta-analysis. PLoS One.

[9] Chandrasekaran RR, Gnanaselane JJ (2008). Predictors of repeat suicidal attempts after first-ever attempt: a two-year follow-up study. Hong Kong J Psychiatry.

[10] Mohamed F, Perera A, Wijayaweera K, Kularatne K, Jayamanne S, Eddleston M (2011). The prevalence of previous self-harm amongst self-poisoning patients in Sri Lanka. Soc Psychiatry Psychiatr Epidemiol.

[11] Knipe D, Metcalfe C, Hawton K, Pearson M, Dawson A, Jayamanne S (2019). Risk of suicide and repeat self-harm after hospital attendance for non-fatal self-harm in Sri Lanka: a cohort study. Lancet Psychiatry.

[12] Gunnell D, Eddleston M (2003). Suicide by intentional ingestion of pesticides: a continuing tragedy in developing countries. Int J Epidemiol.

[13] Yip P, Yousuf S, Chang S-S, Caine E, Chien-Chang Wu K, Chen Y-Y (2012). Means restriction for suicide prevention. Lancet.

[14] Gunnell D, Knipe D, Chang SS, Pearson M, Konradsen F, Lee WJ (2017). Prevention of suicide with regulations aimed at restricting access to highly hazardous pesticides: a systematic review of the international evidence. Lancet Glob Heal.

[15] Chen Ying-Yeh, Wu Kevin Chien-Chang, Wang Yun, Yip Paul S. F. (2016). Suicide Prevention Through Restricting Access to Suicide Means and Hotspots. The International Handbook of Suicide Prevention.

[16] Azrael Deborah, Miller Matthew J. (2016). Reducing Suicide Without Affecting Underlying Mental Health. The International Handbook of Suicide Prevention.

[17] World Health Organization (2014). Preventing suicide: a global imperative.

[18] Zalsman G, Hawton K, Wasserman D, van Heeringen K, Arensman E, Sarchiapone M (2016). Suicide prevention strategies revisited: 10-year systematic review. Lancet Psychiatry.

[19] World Health Organization (2010). The Who recommended classification of pesticides by hazard and guidelines to classification 2009.

[20] Gunnell D, Fernando R, Hewagama M, Priyangika WDD, Konradsen F, Eddleston M (2007). The impact of pesticide regulations on suicide in Sri Lanka. Int J Epidemiol.

[21] Eddleston M (2000). Patterns and problems of deliberate self-poisoning in the developing world. QJM.

[22] Eddleston M, Buckley NA, Eyer P, Dawson AH (2008). Management of acute organophosphorus pesticide poisoning. Lancet.

[23] Verma V, Paul S, Ghose A, Eddleston M, Konradsen F (2017). Treatment of self-poisoning at a tertiary-level hospital in Bangladesh: cost to patients and government. Trop Med Int Heal.

[24] Pearson M, Metcalfe C, Jayamanne S, Gunnell D, Weerasinghe M, Pieris R (2017). Effectiveness of household lockable pesticide storage to reduce pesticide self-poisoning in rural Asia: a community-based, cluster-randomised controlled trial. Lancet.

[25] Knipe Duleeka W., Eddleston Michael (2019). Response to Reifels et al., Suicide and Life‐Threatening Behavior. Suicide and Life-Threatening Behavior.

[26] Dandona Rakhi, Kumar G Anil, Dhaliwal R S, Naghavi Mohsen, Vos Theo, Shukla D K, Vijayakumar Lakshmi, Gururaj G, Thakur J S, Ambekar Atul, Sagar Rajesh, Arora Megha, Bhardwaj Deeksha, Chakma Joy K, Dutta Eliza, Furtado Melissa, Glenn Scott, Hawley Caitlin, Johnson Sarah C, Khanna Tripti, Kutz Michael, Mountjoy-Venning W Cliff, Muraleedharan Pallavi, Rangaswamy Thara, Varghese Chris M, Varghese Mathew, Reddy K Srinath, Murray Christopher J L, Swaminathan Soumya, Dandona Lalit (2018). Gender differentials and state variations in suicide deaths in India: the Global Burden of Disease Study 1990–2016. The Lancet Public Health.

[27] Institute for Health Metrics and Evaluation (IHME) (2018). GBD Compare.

[28] Armstrong G, Vijayakumar L (2018). Suicide in India: a complex public health tragedy in need of a plan. Lancet Public Heal.

[29] Patel V, Ramasundarahettige C, Vijayakumar L, Thakur JS, Gajalakshmi V, Gururaj G (2012). Suicide mortality in India: a nationally representative survey. Lancet.

[30] Arya V, Page A, Gunnell D, Dandona R, Mannan H, Eddleston M (2019). Suicide by hanging is a priority for suicide prevention: method specific suicide in India (2001–2014). J Affect Disordes.

[31] Ahuja H, Mathai AS, Pannu A, Arora R (2015). Acute poisonings admitted to a tertiary level intensive care unit in northern India: patient profile and outcomes. J Clin Diagnostic Res.

[32] Banerjee I, Tripathi SK, Roy AS (2012). Clinico-epidemiological characteristics of patients presenting with organophosphorus poisoning. N Am J Med Sci.

[33] Chaudhary S, Vora SGMDH, Modi P, Chauhan V, Chotaliya D (2013). An epidemiological study of fatal Aluminium phosphide poisoning at Rajkot. IOSR J Pharm.

[34] Gupta SK, Kumar S, Sheikh MI (2006). Study of organophosphorus poisoning in Surat, India. J Indian Acad Forensic Med.

[35] Murali R, Bhalla A, Singh D, Singh S (2008). Acute pesticide poisoning: 15 years experience of a large north-west Indian hospital. Clin Toxicol.

[36] Mathai A, Bhanu MS (2010). Acute aluminium phosphide poisoning: can we predict mortality?. Indian J Anaesth.

[37] Sharma A, Balasubramanian P, Gill KD, Bhalla A (2018). Prognostic significance of blood glucose levels and alterations among patients with aluminium phosphide poisoning. Sultan Qaboos Univ Med J [SQUMJ].

[38] Singh D, Dewan I, Pandey AN, Tyagi S (2003). Spectrum of unnatural fatalities in the Chandigarh zone of north-West India - a 25 year autopsy study from a tertiary care hospital. J Clin Forensic Med.

[39] Singh S, Behera D, Chaudary D, Gupta D, Jindal S (2001). Aggressive atropinisation and continuous pralidoxime (2-PAM) infusion in patients with severe organophosphate poisoning: experience of a northwest Indian hospital. Hum Exp Toxicol.

[40] Srinivas Rao CH, Venkateswarlu V, Surender T, Eddleston M, Nick A (2007). Pesticide poisoning in South India – opportunities for prevention and improved medical management. Trop Med Int Heal.

[41] Chugh S, Dushyant RS, Arora B, Malhotra K (1991). Incidence & outcome of aluminium phosphide poisoning in a hospital study. Indian J Med Res.

[42] Dandona R, Bertozzi-Villa A, Kumar GA, Dandona L (2016). Lessons from a decade of suicide surveillance in India: who, why and how?. Int J Epidemiol.

[43] National Crime Records Bureau (2016). Accidental deaths and suicides in India 2015.

[44] Snowdon J (2019). Indian suicide data: what do they mean?. Indian J Med Res.

[45] Joseph A, Abraham S, Muliyil JP, George K, Prasad J, Minz S (2003). Evaluation of suicide rates in rural India using verbal autopsies, 1994-9. Br Med J.

[46] Prasad J, Abraham VJ, Minz S, Abraham S, Joseph A, Muliyil JP (2006). Rates and factors associated with suicide in Kaniyambadi block, Tamil Nadu, South India, 2000-2002. Int J Soc Psychiatry.

[47] Banerjee S, Chowdhury AN, Schelling E, Weiss MG (2013). Household survey of pesticide practice, deliberate self-harm, and suicide in the sundarban region of West Bengal. India Biomed Res Int.

[48] Joshi R, Guggilla R, Praveen D, Maulik PK (2015). Suicide deaths in rural Andhra Pradesh - a cause for global health action. Trop Med Int Heal..

[49] Gajalakshmi V, Peto R (2007). Suicide rates in rural Tamil Nadu, South India: verbal autopsy of 39000 deaths in 1997-98. Int J Epidemiol.

[50] Vijayakumar L, Jeyaseelan L, Kumar S, Mohanraj R, Devika S, Manikandan S (2013). A central storage facility to reduce pesticide suicides--a feasibility study from India. BMC Public Health.

[51] Vijayakumar L, Satheesh BR (2009). Does “No Pesticide” reduce suicides?. Int J Soc Psychiatry.

[52] Government of India. The Insecticides Act, 1968 (Act No. 46 of 1968). New Delhi; 1968. http://www.krishi.bih.nic.in/Acts-Rules/Insecticides_Act_1968.pdf. Accessed 1 Dec 2019.

[53] Government of India. Insecticide Rules, 1971. New Delhi; 1971. http://www.krishi.bih.nic.in/Acts-Rules/Insecticides_Act_1968.pdf. Accessed 1 Dec 2019.

[54] Chari M. Draft Bill on regulating pesticides could punish farmers who use spurious products, experts fear. Scroll. 2018; https://scroll.in/article/869565/draft-bill-on-regulating-pesticides-could-punish-farmers-who-use-spurious-products-experts-fear. Accessed 5 Sep 2019.

[55] PRS Legislative Research. Legislative Brief - Pesticides Management Bill 2008. 2009. https://prsindia.org/sites/default/files/bill_files/Legislative_Brief-pesticides_management_bill.pdf. Accessed 5 Dec 2019.

[56] PRS Legislative Research (2018). Draft Pesticide Management Bill, 2017.

[57] Standing Committee on Agriculture (2016). Page 104–106 Impact of chemical fertilizers and pesticides on agriculture and allied sectors in the country.

[58] Standing Committee on Agriculture (2016). Page 72–73 Impact of chemical fertilizers and pesticides on agriculture and allied sectors in the country.

[59] Government of Kerala (2011). Banning order of 14 pesticides.

[60] Government of Punjab (2018). Regulation of Sale of Insecticides in Punjab.

[61] South China Morning Post (2018). India’s ‘fully organic’ Sikkim state wins top UN-backed prize for its ecological revolution.

[62] Government of Sikkim. The Sikkim Agricultural, Horticultural Input And Livestock Feed Regulatory Act, 2014: Sikkim Government Gazette; 2015. http://www.lawsofindia.org/pdf/sikkim/2014/2014Sikkim10.pdf. Accessed 26 Nov 2019

[63] Chowdhury FR, Dewan G, Verma VR, Knipe DW, Isha IT, Faiz MA (2017). Bans of WHO class I pesticides in Bangladesh-suicide prevention without hampering agricultural output. Int J Epidemiol.

[64] Cha ES, Sen CS, Gunnell D, Eddleston M, Khang YH, Lee WJ (2016). Impact of paraquat regulation on suicide in South Korea. Int J Epidemiol.

[65] Knipe DW, Sen CS, Dawson A, Eddleston M, Konradsen F, Metcalfe C (2017). Suicide prevention through means restriction: impact of the 2008-2011 pesticide restrictions on suicide in Sri Lanka. PLoS One.

[66] Ministry of Statistics and Programme Implementation (2017). Agriculture - Statistical Year Book India 2017.

[67] The World Bank (2019). World Bank Open Data.

[68] Government of India (2018). Central Insecticides Board & Registration Committee. Directorate of Plant Protection, Quarantine & Storage.

[69] Dawson AH, Eddleston M, Senarathna L, Mohamed F, Gawarammana I, Bowe SJ (2010). Acute human lethal toxicity of agricultural pesticides: a prospective cohort study. PLoS Med.

[70] Government of India (2018). Chemical and Petrochemical Statistics at a Glance - 2018.

[71] Government of India (2013). Chemical and Petrochemical Statistics at a Glance - 2013.

[72] Government of India (2007). Performance of chemical and petrochemical industry at a glance (2001-2007).

[73] Office of the Registrar General & Census Commissioner. 2001 Census Data. Government of India: Ministry of Home Affairs. http://www.censusindia.gov.in/Census_Data_2001/India_at_glance/popu1.aspx. Accessed 5 Sep 2019

[74] Office of the Registrar General & Census Commissioner. 2011 Census Data. Government of India: Ministry of Home Affairs. http://www.censusindia.gov.in/2011-Common/CensusData2011.html. Accessed 5 Sep 2019

[75] Office of the Registrar General & Census Commissioner (2015). SRS Statistical Report 2015.

[76] Mapchart.net. Custom map maker. 2019. https://mapchart.net/. Accessed 5 Sep 2019.

[77] Arya V, Page A, River J, Armstrong G, Mayer P (2018). Trends and socio-economic determinants of suicide in India: 2001–2013. Soc Psychiatry Psychiatr Epidemiol.

[78] National Cancer Institute (2019). Joinpoint Trend Analysis. Division of Cancer Control and Population Sciences.

[79] Prashar A, Ramesh M (2018). Assessment of pattern and outcomes of pesticides poisoning in a tertiary care hospital. Trop Med Int Heal.

[80] StataCorp LLC (2017). Stata statistical software.

[81] Government of Kerala (2011). Substitutes for pesticides banned by Govt of Kerala 7th May 2011.

[82] Government of India. Notification on the Prohibition for Sale, Distribution and Use of Endosulfan in the State of Kerala till further orders. New Delhi: Govt of India Controller of Publications; 2005.

[83] Government of India (2016). Question to the Minister of State in the Ministry of Agriculture and Farmers’ Welfare - Use of Banned Pesticides.

[84] Government of India (2019). Insecticides / Pesticides Registered under section 9(3) of the Insecticides Act, 1968 for use in the Country: (As of 15/05/2019).

[85] Government of India (2019). List of pesticides which are banned, refused registration and restricted in use.

[86] Agropedia (2009). List of banned pesticides in India.

[87] Ministry of Agriculture and Farmer’s Welfare (2018). Pesticides (Prohibition) Order 2018.

[88] Ministry of Agriculture and Farmer’s Welfare (2016). Banning of Pesticides Order 2016.

[89] Ministry of Agriculture and Farmer’s Welfare (2016). October 2016 Inclusion of substances in the Schedule of the Insecticides Act, 1968.

[90] Ministry of Agriculture and Farmer’s Welfare (2017). October 2017 Inclusion of substances in the Schedule of the Insecticides Act, 1968.

[91] Press Information Bureau (2016). Steps to Tackle Banned Pesticides.

[92] Government of India. Minutes of 361st special meeting of registration committee. 2015. http://ppqs.gov.in/sites/default/files/361rc2015.pdf. Accessed 5 Sep 2019.

[93] Singh P, Wakade C, Ambadekar N, Undirwade D, Waghmare V, Deshkar K (2017). Report of special investigation team with regards to poisoning to some and death of some farmers/farm workers during spraying pesticides in Yavatmal District.

[94] All India Network Project on Pesticide Residues. Monitoring of pesticide residues at national level -annual progress report. 2015. http://www.indiaenvironmentportal.org.in/files/file/Annl_rpt_2015.pdf. Accessed 5 Sep 2019.

[95] Agro News (2018). India Punjab Government bans sale of 20 insecticides.

[96] Anya S. State seeks ban on pesticides used by inhalation victims of 2017: The Times of India; 2018. https://timesofindia.indiatimes.com/city/nagpur/state-seeks-ban-on-pesticides-used-by-inhalation-victims-of-2017/articleshow/64034941.cms. Accessed 5 Sep 2019

[97] The Hindu. Karnataka bans use of Endosulfan. The Hindu. 2011; https://www.thehindu.com/news/national/karnataka/Karnataka-bans-use-of-Endosulfan/article15448337.ece#! Accessed 5 Sep 2019.

[98] Hindustan Times (2007). 25 pesticides banned in India.

[99] Chari M, Govindarajan V. Lethal dose: Indian farmers are dying because the government is regulating pesticides poorly. Scroll. 2018; https://scroll.in/article/875775/lethal-dose-indian-farmers-are-dying-because-the-government-is-regulating-pesticides-poorly. Accessed 5 Sep 2019.

[100] Public Eye (2017). The Yavatmal scandal.

[101] Food and Agriculture Organization of the United Nations (FAO) (1997). Parathion.

[102] World Health Organization. Health implications from monocrotophos use : a review of the evidence in India. New Delhi: World Health Organization Regional Office for South-East Asia; 2009.

[103] Sharma BM, Bharat GK, Tayal S, Nizzetto L, Larssen T (2014). The legal framework to manage chemical pollution in India and the lesson from the persistent organic pollutants (POPs). Sci Total Environ.

[104] Pearson M, Zwi AB, Buckley NA, Manuweera G, Fernando R, Dawson AH (2015). Policymaking “under the radar”: a case study of pesticide regulation to prevent intentional poisoning in Sri Lanka. Health Policy Plan.

[105] Knipe DW, Gunnell D, Eddleston M (2015). Preventing deaths from pesticide self-poisoning — learning from Sri Lanka ’ s success. Lancet Glob Heal.

[106] Mi Moon J, Jo CB (2009). Acute endosulfan poisoning: a retrospective study. Hum Exp Toxicol.

[107] Rajapakse T, Griffiths KM, Christensen H (2013). Characteristics of non-fatal self-poisoning in Sri Lanka: a systematic review. BMC Public Health.

[108] Das S, Laya KS (2016). Urbanization and development in Kerala. Int J Appl Res.

[109] Chang SS, Lu TH, Sterne JA, Eddleston M, Lin JJ, Gunnell D. The impact of pesticide suicide on the geographic distribution of suicide in Taiwan: a spatial analysis. BMC Public Health. 2012;12:260. 10.1186/1471-2458-12-260. 10.1186/1471-2458-12-260 PMC3351735 22471759

[110] Mathrubhumi (2018). Endosulfan, other banned pesticides still in use in India.

[111] Joseph S. Banned pesticides: Agriculture department on alert. The Times of India 2017. https://timesofindia.indiatimes.com/city/thiruvananthapuram/banned-pesticides-agriculture-department-on-alert/articleshow/61525455.cms. Accessed 5 Sep 2019.

[112] Radhakrishnan R, Andrade C (2012). Suicide : An Indian perspective. Indian J Psychiatry.

[113] Bhad RM, Hazari N. Reveal rather than conceal: implications of decriminalization of suicide attempts in India. Response to: Bagcchi S, Chaudhuri P. Suicide and the law in India. BMJ. 2015;347:f6975. 10.1136/bmj.f6975 24264769

[114] Government of India (1860). Indian Penal Code.

[115] Ministry of Health and Family Welfare (2018). Notification - Mental Healthcare Act 2017.

[116] Jacob KS (2017). Suicide in India: part perceptions, partial insights, and inadequate solutions. Natl Med J India.

[117] Vijaykumar L (2007). Suicide and its prevention: the urgent need in India. Indian J Psychiatry.

[118] World Health Organization (2018). National suicide prevention strategies.

[119] World Health Organization, Food and Agriculture Organization of the United Nations (FAO) (2019). Preventing suicide - a resource for pesticide registrars and regulators.

[120] Vethanayagam A. V. A. (1962). "Folidol" (Parathion) Poisoning. BMJ.

[121] Federation of Indian Chambers of Commerce and Industry. Indian Agrochemical Industry. http://ficci.in/events/20563/Add_docs/SectorBrief.pdf. Accessed 5 Sep 2019.

[122] Standing Committee on Agriculture (2016). Page 63 Impact of chemical fertilizers and pesticides on agriculture and allied sectors in the country.

[123] Cooper J, Dobson H (2007). The benefits of pesticides to mankind and the environment. Crop Prot.

[124] Singh R, Das KN. Deadly pesticide in temple food that killed 15 in India. Reuters. 2018; https://uk.reuters.com/article/uk-india-foodpoisoning/deadly-pesticide-in-temple-food-that-killed-15-in-india-idUKKBN1OH1LQ. Accessed 5 Sep 2019.

[125] Manuweera G, Eddleston M, Egodage S, Buckley NA (2008). Do targeted bans of insecticides to prevent deaths from self-poisoning result in reduced agricultural output?. Environ Health Perspect.

[126] Barzman M, Bàrberi P, Birch ANE, Boonekamp P, Dachbrodt-Saaydeh S, Graf B (2015). Eight principles of integrated pest management. Agron Sustain Dev.

[127] Van Der Hoek W, Konradsen F, Athukorala K, Wanigadewa T (1998). Pesticide poisoning: a major health problem in Sri Lanka. Soc Sci Med.

[128] Food and Agriculture Organization of the United Nations (FAO), World Health Organization (WHO) (2014). The International Code of Conduct on Pesticide Management.

[129] Krishnankutty K, Hali R, Rajasekharan P, Balachandran P, Suma K, Ajithkumar R (2015). Agricultural Development Policy.

[130] Standing Committee on Agriculture (2016). Page 55 Impact of chemical fertilizers and pesticides on agriculture and allied sectors in the country.

[131] Standing Committee on Agriculture (2016). Page 99 Impact of chemical fertilizers and pesticides on agriculture and allied sectors in the country.

[132] McConnell R, Hruska AJ (1993). An epidemic of pesticide poisoning in Nicaragua: Implicationas for prevention in developing countries. 117th annual meeting of the American public health association (1989, Chicago, Illinois). Am J Public Health.

[133] Konradsen F, Van Der Hoek W, Cole DC, Hutchinson G, Daisley H, Singh S (2003). Reducing acute poisoning in developing countries - options for restricting the availability of pesticides. Toxicology.

[134] United Nations. Sustainable development goal 3 - targets and indicators. Sustainable Development Goals Knowledge Platform. 2019; https://sustainabledevelopment.un.org/sdg3. Accessed 28 Aug 2019.

[135] Hendin H, Phillips MR, Vijayakumar L, Pirkis J, Wang H, Yip P, et al. Suicide and Suicide Prevention in Asia. Geneva: World Health Organisation; 2008.

[136] Roberts DM, Karunarathna A, Buckley NA, Manuweera G, Sheriff MHR, Eddleston M (2003). Influence of pesticide regulation on acute poisoning deaths in Sri Lanka. Bull World Health Organ.

