# Supplementary material for: Suicide by pesticide poisoning in India: a review of pesticide regulations and their impact on suicide trends
Source: BMC Public Health. 2020 Feb 19;20:251. doi: 10.1186/s12889-020-8339-z (PMC7031890; doi:10.1186/s12889-020-8339-z)

# SUPPLEMENTARY MATERIAL

# Supplementary table : timeline of partial restrictions

| **Date** | **Territory** | **Pesticide restricted** |
| --- | --- | --- |
| **1999** | National | methyl bromide ^1^ |
| **2001** | National | **aluminium phosphide** ^2^, captafol ^3^ |
| **2005** | National | **monocrotophos** ^4^ |
| **2007** | National | **fenitrothion** ^5^ |
| **2008** | National | dazomet ^6^ |
| **2009** | National | **cypermethrin** ^1^ |
| **Before 2012 ^b^** | National | **phosphamidon** ^7^, **carbofuran** ^7^, **methomyl** ^7^ |
| **2018** | National | sodium cyanide ^8^, trifluralin ^9^ |

**Restrictions:** 1: restricted to government use only; 2; use of tube packs with 10 or 20 tablets of aluminium phosphide 3 grams banned 3: use as spray banned, seed dresser permitted; 4: use on vegetables not permitted; 5: locust control in desert areas only; 6: use on tea not permitted; 7: only stronger formulations banned; 8: use as insecticide not permitted; 9: use permitted for wheat only.

The following 18 pesticides were refused registration upon application to the Registration Committee: EPN, mevinphos, disulfoton, azinphos-ethyl, azinphos-methyl, calcium arsenate, dicrotophos, lead arsenate, vamidothion, 2,4,5-T, fentin acetate, fentin hydroxide, ammonium sulfamate, chinomethionat, binapacryl, carbophenothion, leptophos, mephospholan.

**^b^:** information on when these pesticides were restricted is not available.

# Supplementary figure legend

**Supplementary figure 1.** Annual pesticide suicides by state from 1995 to 2015

Supplementary figure 2. Map of total number of pesticide suicides by state from 1995 to 2015

**Supplementary figure 3.** Map of change in rate of pesticide suicide by state from 2001 to 2015

**Supplementary figure 4.** Map of change in rate of total suicide by state from 2001 to 2015

Maps were created by the authors using a freely available tool on mapchart.net and are licensed under a Creative Commons Attribution-Share Alike 4.0 International licence [76].

# Supplementary figure 1: annual pesticide suicides by state from 1995 to 2015

Pesticide suicides deaths

# Supplementary figure 2: total number of pesticide suicides by state from 1995 to 2015


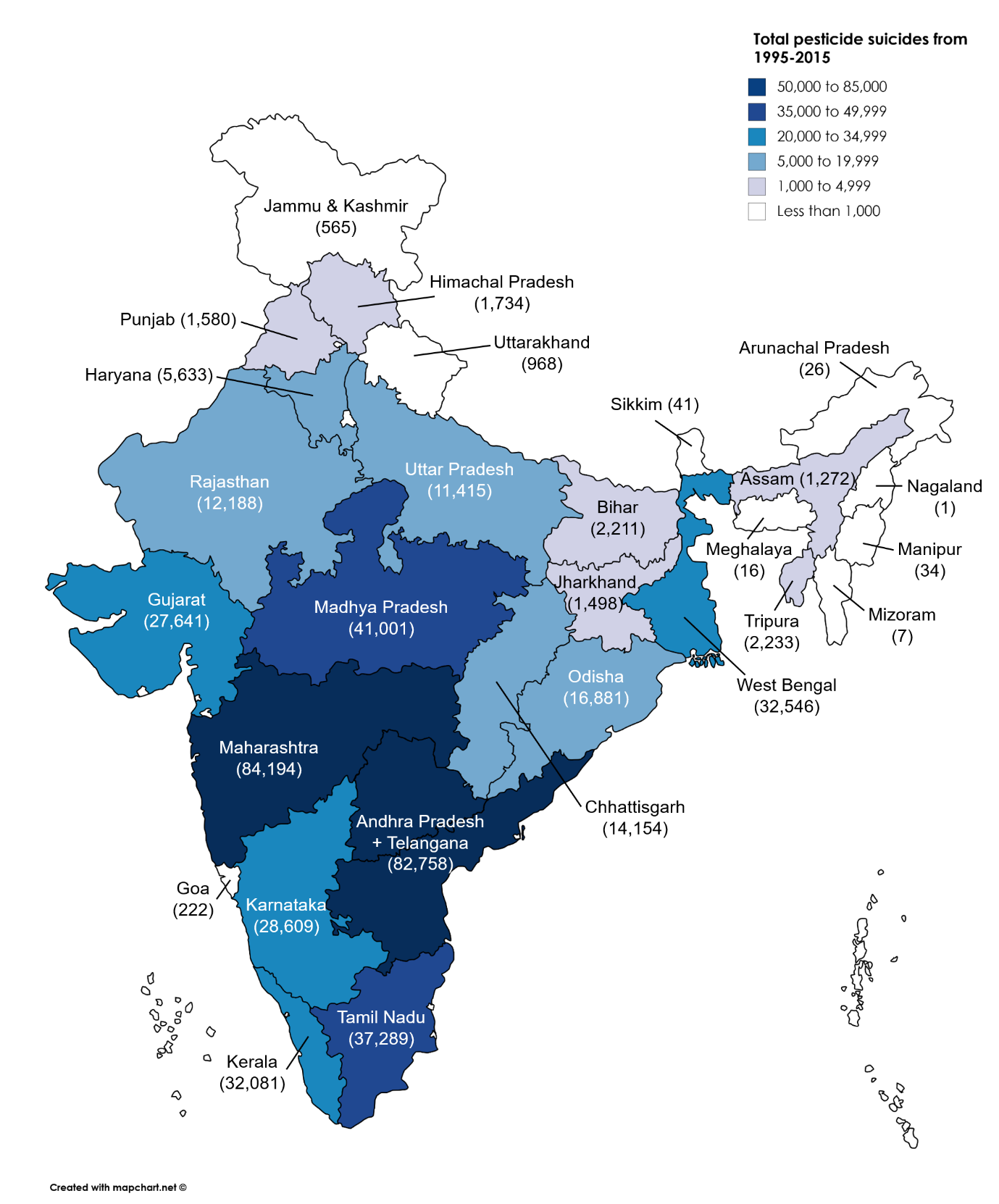


# Supplementary figure 3: map of change in rate of pesticide suicide by state from 2001 to 2015

**
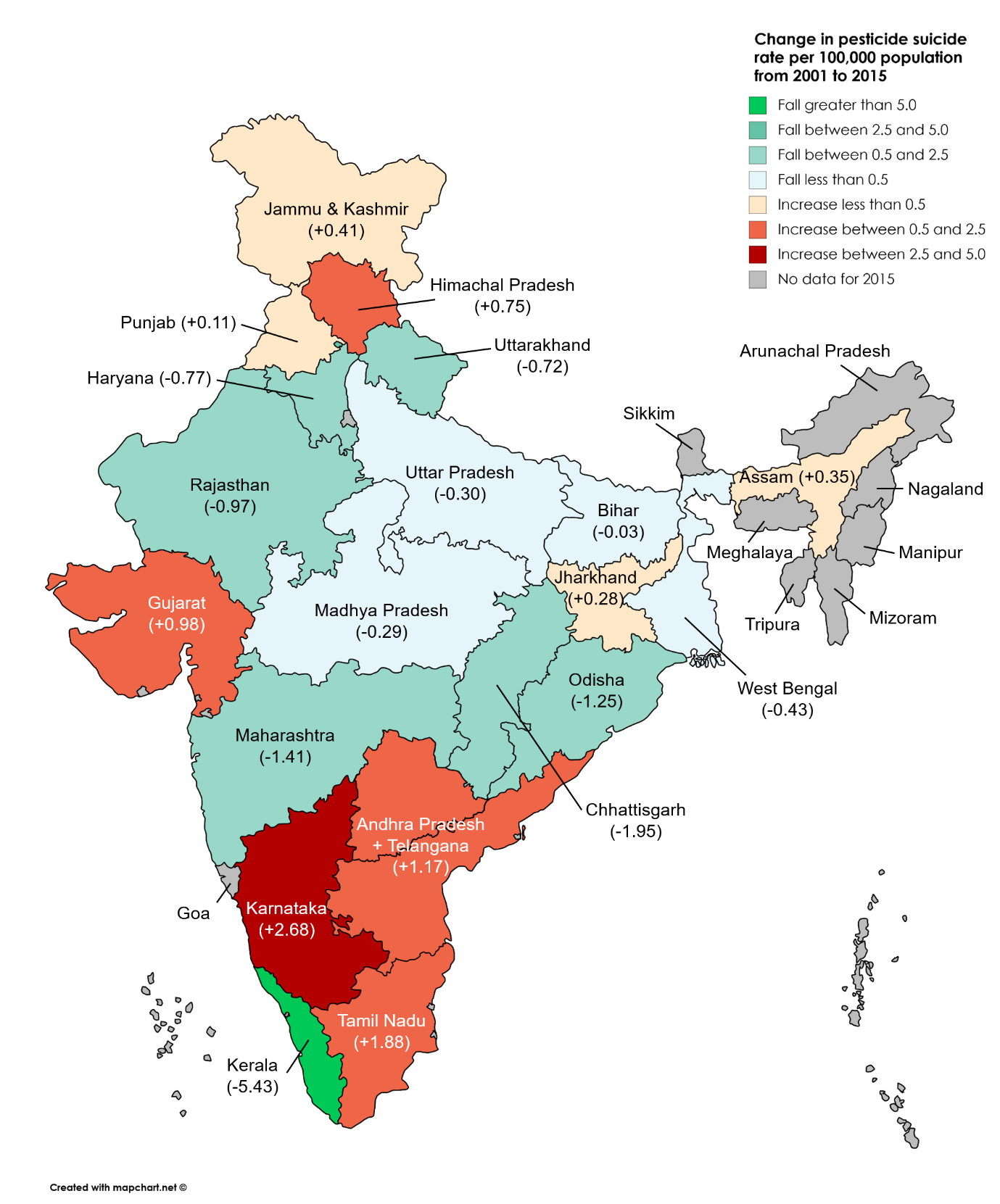
**

# Supplementary figure 4: map of change in rate of total suicide by state from 2001 to 2015


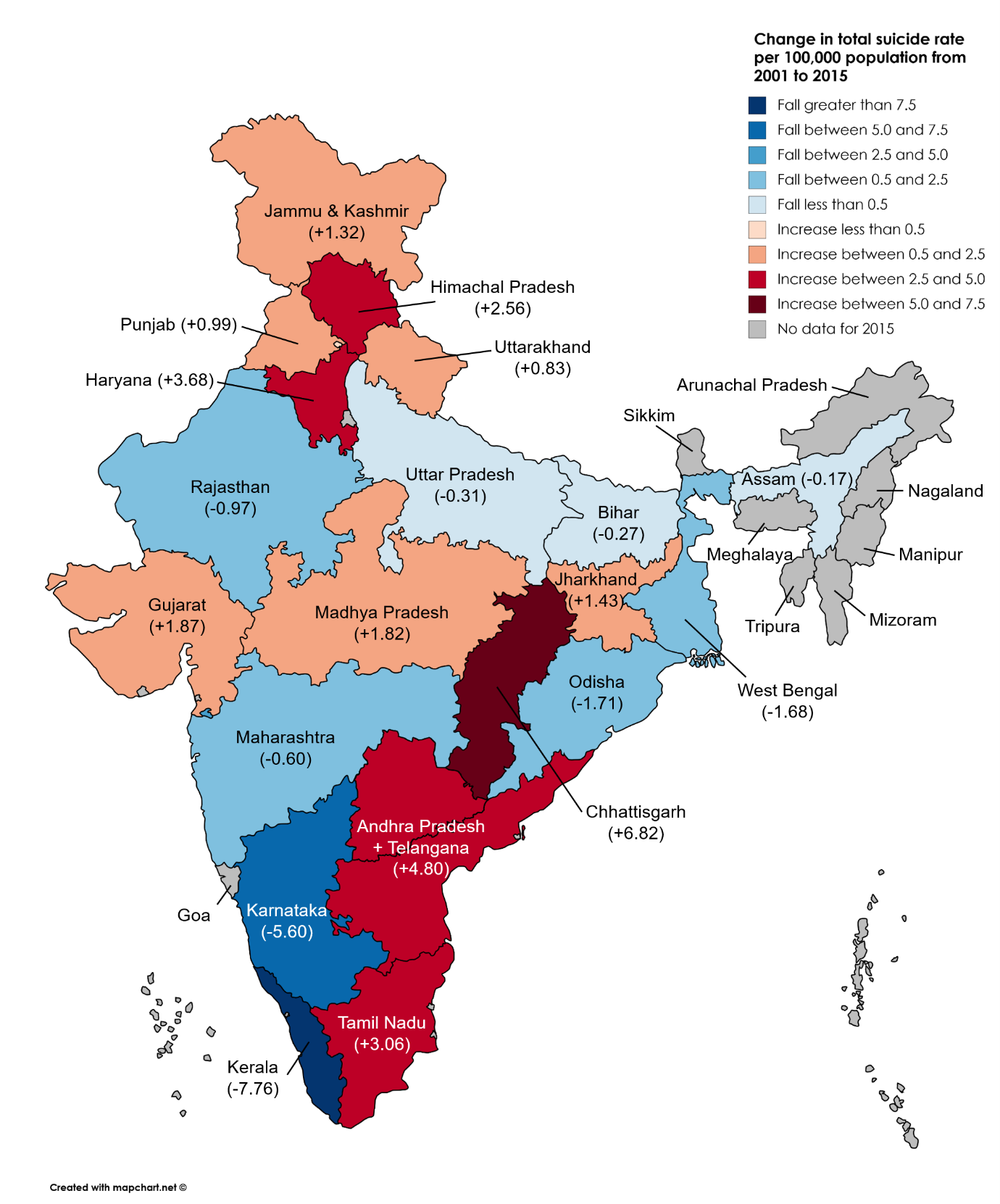

Supplement: Supplementary file 1 — Additional file 1: Table S1. Timeline of partial restrictions. Figure S1. Annual pesticide suicides by state from 1995 to 2015. Figure S2. Map of total number of pesticide suicides by state from 1995 to 2015. Figure S3. Map of change in rate of pesticide suicide by state from 2001 to 2015. Figure S4. Map of change in rate of total suicide by state from 2001 to 2015. [file 12889_2020_8339_MOESM1_ESM.docx]
